# Web-based tools can be used reliably to detect patients with major depressive disorder and subsyndromal depressive symptoms

**DOI:** 10.1186/1471-244X-7-12

**Published:** 2007-04-10

**Authors:** Chao-Cheng Lin, Ya-Mei Bai, Chia-Yih Liu, Mei-Chun Hsiao, Jen-Yeu Chen, Shih-Jen Tsai, Wen-Chen Ouyang, Chia-hsuan Wu, Yu-Chuan Li

**Affiliations:** 1 Department of Psychiatry, National Taiwan University Hospital, Taipei, Taiwan; 2 Graduate Institute of Medical Sciences, Taipei Medical University, Taiwan; 3 Department of Psychiatry, Taipei Veterans General Hospital, Taiwan; 4 Department of Psychiatry, Collage of Medicine, National Yang-Ming University, Taipei, Taiwan; 5 Department of Psychiatry, Chang Gung Memorial Hospital and Chang Gung University, Linkou, Taiwan; 6 Department of Psychiatry, Yuli Veterans Hospital, Hualien, Taiwan; 7 Department of General Psychiatry, Jianan Mental Hospital, D.O.H., Tainan, Taiwan; 8 Department of Psychiatry, Sun Yat-Sen Cancer Center, Taipei, Taiwan; 9 Institute of Biomedical Informatics, National Yang-Ming University, No. 155, Sec. 2, Linong St., Beitou District, Taipei, Taiwan

## Abstract

**Background:**

Although depression has been regarded as a major public health problem, many individuals with depression still remain undetected or untreated. Despite the potential for Internet-based tools to greatly improve the success rate of screening for depression, their reliability and validity has not been well studied. Therefore the aim of this study was to evaluate the test-retest reliability and criterion validity of a Web-based system, the Internet-based Self-assessment Program for Depression (ISP-D).

**Methods:**

The ISP-D to screen for major depressive disorder (MDD), minor depressive disorder (MinD), and subsyndromal depressive symptoms (SSD) was developed in traditional Chinese. Volunteers, 18 years and older, were recruited via the Internet and then assessed twice on the online ISP-D system to investigate the test-retest reliability of the test. They were subsequently prompted to schedule face-to-face interviews. The interviews were performed by the research psychiatrists using the Mini-International Neuropsychiatric Interview and the diagnoses made according to DSM-IV diagnostic criteria were used for the statistics of criterion validity. Kappa (κ) values were calculated to assess test-retest reliability.

**Results:**

A total of 579 volunteer subjects were administered the test. Most of the subjects were young (mean age: 26.2 ± 6.6 years), female (77.7%), single (81.6%), and well educated (61.9% college or higher). The distributions of MDD, MinD, SSD and no depression specified were 30.9%, 7.4%, 15.2%, and 46.5%, respectively. The mean time to complete the ISP-D was 8.89 ± 6.77 min. One hundred and eighty-four of the respondents completed the retest (response rate: 31.8%). Our analysis revealed that the 2-week test-retest reliability for ISP-D was excellent (weighted κ = 0.801). Fifty-five participants completed the face-to-face interview for the validity study. The sensitivity, specificity, positive, and negative predictive values for major depressive disorder were 81.8% and 72.7%, 66.7%, and 85.7% respectively. The overall accuracy was 76.4%.

**Conclusion:**

The evidence indicates the ISP-D is a reliable and valid online tool for assessing depression. Further studies should test the ISP-D in clinical settings to increase its applications in clinical environments with different populations and in a larger sample size.

## Background

Clinical depression is a highly prevalent mental illness. The World Health Organization Global Burden of Disease study in 1997 predicted that clinical depression will be the second most burdensome illness in the world by the year 2020 [1]. The prevalence of depressive symptoms is high, ranging from 20% to 41% of the total population [2,3]. The prevalence of major depressive disorder (MDD) and subsymdormal depressive (SSD) symptoms in an Australian population were found to be 6.8% and 12.9%, respectively [4]. Prevalence of minor depressive disorder (MinD) has been reported to be in the range of 2.6% to 4.5% [5]. Depression is associated with considerably high mortality through suicide, medical illness, and increased risk of accidental death [6]. The high prevalence of depression, together with its significant morbidity, potential treatability, and costs, has made improved detection and management a priority for policymakers and health care agencies [7]. Nevertheless many individuals with depression remain undetected or go untreated [8]. Screening is a frequently proposed strategy for increasing detection of depression; however the diagnostic rate for depression by primary care practitioners is low [9-12]. Many primary care providers fail to diagnose depression in their patients either because they do not know how to screen for depression, have insufficient time, prefer not to label a patient with a mental illness, or do not know how to proceed with a positive result [13]. Furthermore, the current cost of screening for depression may be too high relative to the projected benefit in quality-adjusted life years (QALYs) for many countries to implement. For example, Valenstein et al. found that the cost of screening every 5 years or of one-time screening were $50,988/QALY and $32,053/QALY, respectively [7]. These obstacles impede the progress of screening for depression, which may result in delayed assessment or treatment of the disease. Such delays in diagnosis and treatment can ultimately result in profound consequences for patients including great financial costs, and disruption of interpersonal relationships and employment, as well as diminished overall mental and physical health. For these reasons, it is important to consider programs that can assess risk of depression in a manner that is easier, less costly, and more confidential than the face-to-face interview.

Computer-administered depression assessment programs, such as the computer-administered version of the Hamilton Depression Rating Scales [14], the computerized version of the Center for Epidemiological Studies Depressed Mood Scale (CES-D) [15], the computer-adaptive test for depression (D-CAT) [16], and the electronic version of Mini-International Neuropsychiatric Interview (M.I.N.I.) [17] have been developed in recent years. They give standardized information about a patient's psychopathology [18], and could reduce both the time and the tremendous financial cost of processing and analyzing diagnostic data [19]. Previous researches have indicated that patients preferred the computer-mediated to the clinician interviews for assessment in sensitive areas such as suicide, substance abuse, sexual behavior, and HIV-related symptoms. Patients felt less embarrassed, more relaxed, and were generally more honest when using computer assessments [20-23]. Despite the wealth of encouraging research, few computer aids are being used in regular mental health care in nonresearch settings. One of the reasons for this may be that computer-administered programs still require that people come to a clinical setting to take the test.

The Internet provides advantages that may greatly improve the utility of computer-administered diagnostic programs. Firstly, Web-based programs are an anonymous method of accessing information about socially "unacceptable" illnesses [19]. Furthermore Internet programs can reach a large number of individuals across a broad geographic area at a low cost, and may also identify many persons who have never received treatment [24]. Individuals may surf the Internet conveniently to find information about whether they have depression or whether they should seek treatment. There are several programs through which people may be assessed for depression through the Internet, including an Internet-based depression screening test adapted from the CES-D scale [24] and the Web-Based Depression and Anxiety Test (WB-DAT) [25].

Because they are anonymous, Internet studies are difficult to perform. As yet, only one study has made an attempt to evaluate the validity of a Web-based instrument for depression [25]. In that study, the researchers reported that the WB-DAT had good diagnostic concordance with Structured Clinical Interview for DSM-IV Axis I Disorders (SCID-I/P) diagnosis for MDD. However, the participants in the sample were recruited from several clinical research projects, a subject population that is not representative of Internet users at large. No previous academic reports could be found on the reliability of such programs. In the present study, we describe the design of the Internet-based Self-assessment Program for Depression (ISP-D) and evaluate the test-retest reliability and criterion validity of the ISP-D.

## Methods

### System and program design

The ISP-D was developed by a committee consisting of 16 certified psychiatrists in Taiwan. Its aim is to provide Internet users a concise, interactive, and individualized tool to self-assess depressive symptoms and screen for depressive disorders. It included 3 depression diagnoses: MDD, MinD, and SSD. These three diagnoses were chosen because they include the same nine symptoms and they represent different levels of depression severity. MDD was defined in the DSM-IV criteria [26]. MinD was defined in accordance with the DSM-IV research criteria, that is, an episode with depressed mood or decreased interest, fulfilling at least two (but fewer than five) A criteria for a major depressive disorder [26,27]. SSD was used to evaluate subthreshold depression, which was defined as having two or more symptoms of depression criteria excluding the A criteria [27-29]. The presence of each symptom was determined using one to three questions, which were adapted primarily from the M.I.N.I. [17], and Taiwanese Depression Questionnaire (TDQ) [30]. Some revisions were made to improve understanding and readability on the Internet browser, e.g. more explanations or examples were included and different font colors or styles were incorporated to highlight important messages. Because the program was designed with branching, different users may get different numbers of questions (9 to 24) during a complete assessment.

The interactive ISP-D system was hosted on a Microsoft Windows 2000 server of PsychPark [31] in Taiwan and developed with Active Server Page 3.0 and HTML 3.0. The database system was constructed with a Microsoft SQL server 2000. Visual Basic (VB) script was used for interactive program and real-time data analysis to confirm that all questions had been completed. The Web site and Simple Mail Transfer Protocol (SMTP) service were hosted on Internet Information Services (IIS) 5.0. The transmission of account/password was protected by Secure Sockets Layer on the server. PsychPark, one of the largest and most popular mental health websites among the Chinese population, was established in 1995 as a virtual organization. PsychPark is free and membership is open to the general population.

### Participants

Participants were volunteers recruited via the Internet. They found the study advertisement on PsychPark, which was listed on the Web resources of major portal websites, such as Yahoo and MSN. During the recruitment period, weekly electronic papers were sent to the subscribers of PsychPark e-paper. Individuals who wanted to know whether they had depressive disorders or wanted to recheck their existing depressive disorders were encouraged to join the study [32]. The only inclusion criterion was that participants must be at least 18 years of age because the program was not designed to assess adolescent depression. Participants were excluded if they could not complete the study protocol. The study was approved by the Institute Review Board of the Yuli Veterans Hospital, Taiwan and was performed in compliance with the Helsinki Declaration.

### Procedure

Recruitment for the ISP-D test-retest reliability study was performed from September 2001 to January 2002. The entire process of the reliability study was completed via the Internet. When a participant entered the study website, the program began with an introduction about the aims and the procedure of the study. Each participant had to set up a PsychPark user account and password online to participate in the study, and was asked to visit a real clinic as soon as possible if he/she faced a severe mental condition. After reading a complete description of the study on the browser, the participant signed an informed consent form. Each participant was then asked questions on his or her sociodemographic data. There were brief instructions on how to answer subsequent questions about depressive symptoms and related conditions. We encouraged the participants to answer the questions honestly so that their assessment would achieve more accurate results.

**Figure 1:**
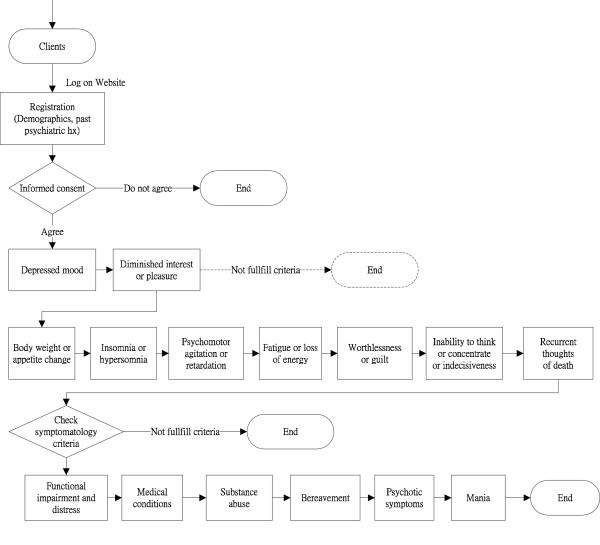
**Algorithm for Internet-based Self-assessment Program for Depression**. Dashed line indicates that the function was not implemented until the study was completed.

The algorithm for performing the ISP-D test is shown in Figure 1. Most of the questions had multiple corresponding choices of answers and participants simply clicked to choose their answer (Additional File 1). Total interview times were recorded. About one to two weeks after the completion of the test, we sent follow-up e-mail messages to notify the participants to perform the ISP-D test again. We sent another e-mail message to respondents that had not performed the retest within 2 weeks of the first follow-up email notice. Participants' identifying PsychPark accounts precluded them from repeating the ISP-D test without permission before the minimal re-test interval.

After completing the self-administered assessments, the participants were asked to make an appointment with a psychiatrist of our research team near his/her location for the validity study. Our research team included 16 certified psychiatrists who were located in most of the geographic areas of Taiwan. The psychiatrists had been trained to use the M.I.N.I. [17], a clinical diagnostic interview based on the DSM-IV. They performed face-to-face semi-structured interviews with the M.I.N.I. and made psychiatric diagnoses according to the DSM-IV. Recruitment for the validity study was performed from September 2001 to May 2002 [33].

### Data analysis

Subjects were classified as having MDD, MinD, SSD, or as no depression specified (ND). The system's test-retest reliability was analyzed by computing weighted kappa (κ) statistics for various time intervals. In order to examine the test-retest reliability for each diagnosis, κ values were recalculated for the groupings of MDD vs. non-MDD, MinD vs. non-MinD, and SSD vs. non-SSD. The test-retest reliability for each depressive symptom was calculated using the same method. To analyze the representation of our retest sample, sociodemographic data were compared between the retest responders and non-responders. To analyze the variables affecting test-retest agreement, we compared the sociodemographic data between participants with test-retest agreement and those without agreement.

The psychiatrist's diagnosis was regarded as the criterion for the validity statistics. Validity statistics included sensitivity, specificity, positive predictive value (PPV), and negative predictive value (NPV). To analyze the representation of our validity sample, sociodemographic data were compared between participants entering the validity study (validity sample) and those not entering the validity study (non-validity sample). To analyze the variables affecting the agreement between the diagnoses of ISP-D and psychiatrists, we compared the sociodemographic data between participants with agreement and those without agreement.

A Chi-square test was used to compare categorical variables and t-tests were used to compare continuous variables between group pairs. Standard deviations were calculated for all mean values. A p value of <0.05 (two-tailed) was considered significant for all statistical tests. The statistical software used was SPSS 10.0 for Windows. Weighted kappa was analyzed with the SAS System 9.0 for Windows.

## Results

### Test-retest reliability study

There were 579 valid participants who completed the ISP-D test. Their demographic data and characteristics are summarized in Table 1. The mean age of the study population was 26.5 ± 6.6 years and their mean education level was 14.9 ± 2.1 years. Most of the participants were female (72.7%), and single (82%). The distributions of MDD, MinD, SSD, and ND were 30.9% (n = 179), 7.4% (n = 43), 15.2% (n = 88), and 46.5% (n = 269), respectively. The mean Internet interview time was 8.89 ± 6.77 min.

**Table 1:** Demographic data for the ISP-D test, retest, and validity samples

**Variables**	**Test sample (n = 579)**	Retest sample (n = 184)*	**Validity sample (n = 55)† **
**Age (years) **		*NS*	*P < 0.001*
Mean ± SD	26.5 ± 6.6	26.2 ± 6.6	29.7 ± 7.5
Range	18–52	18–49	18–51
**Education (years)**		*NS*	*NS*
Mean ± SD	14.9 ± 2.1	15.2 ± 1.9	15.3 ± 2.0
Range	0–21	9–21	9–18

	%	%	%

**Sex**		*NS*	*NS*
Female	72.7	77.7	76.4
Male	27.3	22.3	23.6
**Marital status**		*NS*	*NS*
Married	18	18.5	23.6
Single	82	81.5	76.4
**Employment status**		*NS*	*P = 0.027*
Employed	51.8	48.4	67.3
Student	39.4	44.6	23.6
Unemployed	8.8	7.1	9.1

* Statistical comparisons were made between retest responders and non-responders. All p values > 0.05.

† Statistical comparisons were made between validity responders and non-responders. P value was shown when p < 0.05.

*NS*: no significant statistical difference.

ISP-D: Internet-based Self-assessment Program for Depression.

One hundred and eighty-four of the respondents completed the retest (response rate: 31.8%). Their diagnostic distributions of MDD, MinD, SSD, and ND were 39.1% (n = 72), 8.7% (n = 16), 22.3% (n = 41), and 29.9% (n = 55), respectively. Sociodemographic data between the retest responders and non-responders did not differ. The test-retest reliability results calculated by weighted κ were shown in Table 2 for each of the following time intervals: within 2 weeks, between 2 and 4 weeks, and greater than 4 weeks. The overall test-retest reliability was excellent (weighted κ = 0.801) within 2 weeks. The κ values for each diagnosis within 2 weeks, between 2 and 4 weeks, and greater than 4 weeks were: MDD (0.830, 0.449, and 0.499), MinD (0.029, 0.197, and 0.316), and SSD (0.812, 0.329, and 0.466). The test-retest reliabilities for each depressive symptom are shown in Table 3. The test-retest reliability for each depressive symptom was fair to excellent within a 2-week interval. κ is considered to reflect the agreement beyond chance. A κ value of at least 0.75 is regarded as indicative of excellent levels of agreement, 0.60–0.74 as good, 0.40–0.59 as fair, and under 0.40 as poor agreement [34]. The mean test-retest interval was 21.8 ± 16.7 days. There were no statistically significant differences for age, sex, marital status, years of education, employment, test-retest time interval, or testing time between participants with test-retest agreement and those without agreement.

**Table 2:** ISP-D test-retest diagnosis reliabilities by test-retest time interval

**(A)Within 2 weeks of test-retest time interval**					
	**ND**	**SSD**	**MinD**	**MDD**	**Total no.**

**ND**	12	2	0	0	14
**SSD**	0	8	0	1	9
**MinD**	3	0	0	2	5
**MDD**	1	0	1	29	31
**Total no.**	16	10	1	32	59
**Weighted κ**	0.801				

**(B) Between 2 and 4 weeks of test-retest time interval**					

	**ND**	**SSD**	**MinD**	**MDD**	**Total no.**

**ND**	21	4	2	4	31
**SSD**	7	9	2	5	23
**MinD**	2	1	2	2	7
**MDD**	5	2	2	16	25
**Total no.**	35	16	8	27	86
**Weighted κ**	0.432				

**(C) Greater than 4 weeks of test-retest time interval**					

	**ND**	**SSD**	**MinD**	**MDD**	**Total no.**

**ND**	4	4	0	2	10
**SSD**	2	7	0	0	9
**MinD**	1	1	2	0	4
**MDD**	0	3	4	9	16
**Total no.**	7	15	6	11	39
**Weighted κ**	0.509				

ISP-D: Internet-based Self-assessment Program for Depression; MDD: major depressive disorder; MinD: minor depressive disorder; SSD: subsyndromal depressive disorder; ND: no depression specified.

**Table 3:** ISP-D test-retest reliabilities for depressive symptoms by test-retest time interval

**Depressive symptoms**	**κ (agreement) by time interval**		
	
	*<2 weeks (N = 59)*	*≥2 to ≤4 weeks (N = 86)*	*>4 weeks (N = 39)*
**Depressed mood**	0.458 (72.9%)	0.360 (73.3%)	0.412 (71.8%)
**Diminished interest or pleasure**	0.759 (88.1%)	0.557 (77.9%)	0.438 (71.8%)
**Decreased appetite**	0.551 (79.7%)	0.622 (83.7%)	0.206 (59.0%)
**Insomnia or hypersomnia**	0.594 (79.7%)	0.541 (77.9%)	0.584 (79.5%)
**Activity disturbance**	0.433 (72.9%)	0.422 (70.9%)	0.227 (61.5%)
**Fatigue or loss of energy**	0.791 (93.2%)	0.544 (77.9%)	0.379 (76.9%)
**Worthlessness or excessive guilt**	0.423 (71.2%)	0.564 (79.1%)	0.734 (87.2%)
**Diminished ability to think or concentrate **	0.568 (84.7%)	0.546 (79.1%)	0.554 (79.5%)
**Thoughts of death**	0.829 (91.5%)	0.334 (70.9%)	0.556 (79.5%)

ISP-D: Internet-based Self-assessment Program for Depression.

The presence of depressive symptoms was defined as fulfilling the symptomatic criteria of DSM-IV

### Validity study

Fifty-five participants completed the validity study (1076 ISP-D tests during the study period). Their demographic characteristics and a comparison between validity sample and non-validity sample are presented in Table 1. The participants in the validity sample were significantly older than those in the non-validity sample and were more likely to be employed. The interval between the last ISP-D test and the psychiatric interview was 5.7 ± 3.9 days. The mean Internet interview time was 10.29 ± 8.11 min, and the mean face-to-face psychiatric interview time was 22.4 ± 10.7 min. The length of the psychiatric interview was significantly greater than that of the Internet interview (paired-t test, t = 6.97, df = 49, p < 0.001). Because psychiatrists diagnosed only one participant with MinD and only one participant with SSD, it was not possible to calculate the validity for MinD and SSD. Therefore, these two participants were classified as non-MDD and only the validity of MDD was calculated. A comparison of the ISP-D diagnoses and psychiatrists' diagnoses is shown in Table 4. Using the psychiatrist's diagnosis as the criterion, the ISP-D's accuracy was 76.4%, sensitivity 81.8%, specificity 72.7%, PPV 66.7%, and NPV 85.7%. There were no statistically significant differences in the sociodemographic data between participants with diagnostic agreement and those without agreement.

**Table 4:** Comparison of ISP-D diagnoses and psychiatrists' diagnoses for major depressive disorder

**ISP-D diagnoses**	**Psychiatrists' diagnosis**		**Total**
		
	*Depressed*	*Not depressed*	
**Yes **	18	9	27
**No**	4	24	28
**Total**	22	33	55

ISP-D: Internet-based Self-assessment Program for Depression

Sensitivity = 81.8%; Specificity = 72.7%; Positive predictive value = 66.7%; Negative predictive value = 85.7%.

## Discussion

In our analysis, the ISP-D was found to be a reliable and valid tool for Internet users. The reliability and validity of data collected through the Internet have been questioned because the data were based on information provided directly by the patient's experience and data gathering were administered remotely. In the study, the overall test-retest reliability for ISP-D was excellent within a 2-week interval. Regarding the test-retest reliability for each diagnosis within a 2-week interval, MDD and SSD were found to be excellent. Only the reliability of MinD was low, which may be related to the relatively fewer number of cases of MinD. It is possible that MinD was not a stable diagnosis or that it may have been a stage of another depressive disorder. The high reliability of SSD implied that its diagnosis is worthy of further studies of validity and psychopathology. As the test-retest interval increased to greater than 2 weeks, the test-retest reliabilities of MDD and SSD decreased. Because MinD has similar symptomatic criteria to MDD and SSD, the good reliabilities of MDD and SSD lessened the possibility of problems with the questionnaire design or the media bias of the Internet.

The analysis of test-retest reliability for each depressive symptom showed that the reliabilities for "diminished interest or pleasure", "fatigue or loss of energy", and "thoughts of death" were excellent and that those for other symptoms were fair within a 2-week interval. The reliabilities were poor only for "depressed mood" and "thoughts of death" with a time interval between 2 and 4 weeks, and were poor only for "decreased appetite", "activity disturbance", and "fatigue or loss of energy" with a time interval greater than 4 weeks. The test-retest reliability with a time interval of greater than 2 weeks may be affected by the fluctuating course of depressive disorders themselves. According to Judd et al. [35]., the symptomatic course of MDD is dynamic and changeable, and MDD, MinD, and SSD symptom levels commonly alternate over time in the same patients as a symptomatic continuum of illness activity of a single clinical disease. Cuijpers and Smit found that the incidence of MDD in subjects with SSD is larger than in subjects without SSD [36]. Thus in spite of a possible fluctuating course, the test-retest reliability of each depressive symptom in the ISP-D was fair to excellent within a 2-week interval.

For the validity study, the M.I.N.I. was applied in the clinical interview. The concordance between the M.I.N.I. and SCID-P diagnoses was demonstrated to be very good (sensitivity 96%, specificity 88%, PPV 87%, and NPV 97%) [17]. κ values of inter-rater reliability for the M.I.N.I. and for test-retest reliability of MDD were previously reported to be 1.00 and 0.87, respectively [17], indicating that the M.I.N.I. is a good criterion for validity. As of yet, only one study has previously attempted to evaluate the validity of a Web-based instrument for depression diagnosis [25]. In that study Farvolden et al. showed that the WB-DAT had sensitivity, specificity, PPV, and NPV of 79%, 89%, 75%, and 93%, respectively, relative to SCID-I/P diagnosis for MDD [25]. Our result is similar to that of Farvolden et al. and confirms that the Internet may be a valid tool for the assessment of depression. Farvolden et al.'s study differed from the present study in that the participants in their study were recruited from several clinical research projects and the test was performed in a clinical environment. By contrast, our study participants were recruited remotely, directly from the Internet, and the ISP-D was used to evaluate depressive disorders with differing severities including MDD, MinD, and SSD. All the other validity studies for depression have been conducted in writing (pen and paper tests). One such study by Haringsma et al. [37] used the same assessment tool as that used in our study (the M.I.N.I.) to assess the criterion validity of the CES-D in a sample of self-referred seniors. They found that with the optimal cut-off score of 25 for MDD, sensitivity was 85%, specificity 64%, and PPN 63%. In Bagby's review article [38], the mean sensitivity, specificity, PPV, and NPV of the Hamilton Depression Rating Scale from 7 studies were found to be 76%, 91%, 77%, and 92%, respectively. In Nyklicek's study [39] of the Edinburgh Depression Scale (EDS) with 951 randomly selected women of peri-menopausal age, test sensitivity, specificity, PPV, and NPV were 58.8%, 95.0%, 49.1%, and 91.7%, respectively, with a cut-off score of 12. The results of our study demonstrated that screening for depression via the Internet may have similar validity as screening tests conducted in writing, most of which have been reported to be highly sensitive and specific [40].

Despite the satisfying results of the present examination of the reliability and validity of the ISP-D, the study has several limitations. The first limitation of our study is the potential for a self-selected effect. The high prevalence of MDD, MinD, and SSD in the study may be due to a self-selected effect of the participants. That is, people who had depressive symptoms may have been more motivated to participate in the study than non-depressed persons in the general population. On the other hand, many severely depressed patients may not have access to the Internet. The self-selected effect was apparent in the demographic characteristics of our study sample, with the majority of participants being young, single, well educated women. This sampling bias limits our ability to generalize our findings to the general population. The second limitation is the relatively low response rate in the test-retest reliability study, a characteristic inherent to Internet studies [41,42]. Importantly, our statistical analysis showed that the sociodemographic characteristics did not affect the agreement of the test-retest reliability or the validity of the ISP-D, despite these limitations. The third limitation is that the kappa statistics for each diagnosis was recoded and recalculated. The interpretation should be cautious because of possible recoding bias.

Because the mean Internet interview time for the ISP-D was relatively short, and participants did not need to go to a clinic, the ISP-D may be useful as an auxiliary tool for screening or follow-up of depression as an alternative to the face-to-face interview. There is little additional cost to online interviews beyond maintenance of the system on the server. Computer-aided interviewing gives standardized information about a patient's psychopathology and diagnosis. It allows patients to work at their own pace and is available whenever a computer terminal is available. Furthermore, results can be scored and presented to the patients and/or clinicians immediately. Indeed the standardized manner of administration and scoring of computer-administered rating scales may actually improve reliability and insure greater completeness of the information gathered [18].

The ISP-D can generate personalized reports, which summarize each individual's responses and possible diagnostic categories. The automatically generated final report was designed to be printed and shared with a health care professional. In addition, links to related Internet articles and information about resources such as clinics and hospitals, virtual clinics, and online groups can be provided when participants have positive findings.

## Conclusion

To our knowledge, this study was the first to investigate both the reliability and validity of a Web-based depression program. We found that the test-retest reliability of the ISP-D was excellent within a 2-week interval and its criterion validity was comparable to that observed with written tests. The current study offers Internet users an alternative way to assess depression by oneself in a short amount of time. The ISP-D provides a continuously available, inexpensive, and easily maintained depression screening method that is accessible to a large number of individuals across a broad geographic area. In order to broaden the application of the ISP-D, further studies conducted with various populations (outpatients, inpatients etc.) in larger sample sizes (especially for validity) should be conducted.

## Competing interests

The author(s) declare that they have no competing interests.

## Authors' contributions

CCL participated in the system design and programming of the ISP-D, the literature review, the statistical analysis, the interpretation of the findings and the preparation of the manuscript. YCL participated in the study design, the interpretation of the findings and refinement of the manuscript. YMB and CYL participated in the study design, the administration of clinical validation and refinement of the manuscript. MCH, JYC, SJT, WCO, and CHW helped in the design of the questionnaire, administration of the survey and the clinical validation. All authors have read and approved the final manuscript.

## Pre-publication history

The pre-publication history for this paper can be accessed here:



## Supplementary Material

Additional file 1 English translation of questions in the Internet-based Self-assessment Program for Depression (ISP-D). The English translation of ISP-D is preliminary and is intended for the ease of journal readers to understand the original Chinese version of ISP-D. Click here for file

## References

[B1] Murray CJ, Lopez AD (1997). Alternative projections of mortality and disability by cause 1990-2020: Global Burden of Disease Study. Lancet.

[B2] Zung WW, Broadhead WE, Roth ME (1993). Prevalence of depressive symptoms in primary care. J Fam Pract.

[B3] Crum RM, Cooper-Patrick L, Ford DE (1994). Depressive symptoms among general medical patients: prevalence and one- year outcome. Psychosom Med.

[B4] Goldney RD, Fisher LJ, Dal GE, Taylor AW (2004). Subsyndromal depression: prevalence, use of health services and quality of life in an Australian population. Soc Psychiatry Psychiatr Epidemiol.

[B5] Hermens ML, van Hout HP, Terluin B, van der Windt DA, Beekman AT, van DR, de HM (2004). The prognosis of minor depression in the general population: a systematic review. Gen Hosp Psychiatry.

[B6] Fawcett J (1993). The morbidity and mortality of clinical depression. Int Clin Psychopharmacol.

[B7] Valenstein M, Vijan S, Zeber JE, Boehm K, Buttar A (2001). The cost-utility of screening for depression in primary care. Ann Intern Med.

[B8] Davidson JR, Meltzer-Brody SE (1999). The underrecognition and undertreatment of depression: what is the breadth and depth of the problem?. J Clin Psychiatry.

[B9] Joukamaa M, Lehtinen V, Karlsson H (1995). The ability of general practitioners to detect mental disorders in primary health care. Acta Psychiatr Scand.

[B10] Rost K, Zhang M, Fortney J, Smith J, Coyne J, Smith GR (1998). Persistently poor outcomes of undetected major depression in primary care. Gen Hosp Psychiatry.

[B11] Cohen-Cole SA, Boker J, Bird J, Freeman AM (1982). Psychiatric education for primary care: a pilot study of needs of residents. J Med Educ.

[B12] Schulberg HC, Burns BJ (1988). Mental disorders in primary care: epidemiologic, diagnostic, and treatment research directions. Gen Hosp Psychiatry.

[B13] Klinkman MS (2003). The role of algorithms in the detection and treatment of depression in primary care. J Clin Psychiatry.

[B14] Kobak KA, Reynolds WM, Griest JH (1994). Computerized and clinician assessment of depression and anxiety: respondent evaluation and satisfaction. J Pers Assess.

[B15] Ogles BM, France CR, Lunnen KM, Bell MT, Goldfarb M (1998). Computerized depression screening and awareness. Community Ment Health J.

[B16] Fliege H, Becker J, Walter OB, Bjorner JB, Klapp BF, Rose M (2005). Development of a computer-adaptive test for depression (D-CAT). Qual Life Res.

[B17] Sheehan DV, Lecrubier Y, Sheehan KH, Amorim P, Janavs J, Weiller E, Hergueta T, Baker R, Dunbar GC (1998). The Mini-International Neuropsychiatric Interview (M.I.N.I.): the development and validation of a structured diagnostic psychiatric interview for DSM-IV and ICD-10. J Clin Psychiatry.

[B18] Cawthorpe D (2001). An evaluation of a computer-based psychiatric assessment: evidence for expanded use. Cyberpsychol Behav.

[B19] Lissman TL, Boehnlein JK (2001). A critical review of internet information about depression. Psychiatr Serv.

[B20] Black DW, Belsare G, Schlosser S (1999). Clinical features, psychiatric comorbidity, and health-related quality of life in persons reporting compulsive computer use behavior. J Clin Psychiatry.

[B21] Locke SE, Kowaloff HB, Hoff RG, Safran C, Popovsky MA, Cotton DJ, Finkelstein DM, Page PL, Slack WV (1994). Computer interview for screening blood donors for risk of HIV transmission. MD Comput.

[B22] Erdman HP, Klein MH, Greist JH, Skare SS, Husted JJ, Robins LN, Helzer JE, Goldring E, Hamburger M, Miller JP (1992). A comparison of two computer-administered versions of the NIMH Diagnostic Interview Schedule. J Psychiatr Res.

[B23] Slack WV, Hicks GP, Reed CE, Van Cura LJ (1966). A computer-based medical-history system. N Engl J Med.

[B24] Houston TK, Cooper LA, Vu HT, Kahn J, Toser J, Ford DE (2001). Screening the public for depression through the Internet. Psychiatr Serv.

[B25] Farvolden P, McBride C, Bagby RM, Ravitz P (2003). A Web-based screening instrument for depression and anxiety disorders in primary care. J Med Internet Res.

[B26] American Psychiatric Association (1994). Diagnostic and Statistical Manual of Mental Disorders. 4th ed ,.

[B27] Rapaport MH, Judd LL (1998). Minor depressive disorder and subsyndromal depressive symptoms: functional impairment and response to treatment. J Affect Disord.

[B28] Judd LL, Akiskal HS, Paulus MP (1997). The role and clinical significance of subsyndromal depressive symptoms (SSD) in unipolar major depressive disorder. J Affect Disord.

[B29] Sadek N, Bona J (2000). Subsyndromal symptomatic depression: a new concept. Depress Anxiety.

[B30] Lee Y, Yang MJ, Lai TJ, Chiu NM, Chau TT (2000). Development of the Taiwanese Depression Questionnaire. Chang Gung Med J.

[B31] (2007). PsychPark. http://www.psychpark.org/.

[B32] (2007). Internet-based Self-assessment Program for Depression (ISP-D, in traditional Chinese). http://www.psychpark.org/guideline/depression/test/.

[B33] Lin C, Li YC, Bai YM, Tsai SJ, Hsiao MC, Wu CH, Liu CY, Chen JY (2003). The Validity of an Internet-based Self-assessment Program for Depression. Proc AMIA Symp.

[B34] Cicchetti DV, Sparrow SA (1981). Developing criteria for establishing interrater reliability of specific items: applications to assessment of adaptive behavior. Am J Ment Defic.

[B35] Judd LL, Akiskal HS, Maser JD, Zeller PJ, Endicott J, Coryell W, Paulus MP, Kunovac JL, Leon AC, Mueller TI, Rice JA, Keller MB (1998). A prospective 12-year study of subsyndromal and syndromal depressive symptoms in unipolar major depressive disorders. Arch Gen Psychiatry.

[B36] Cuijpers P, Smit F (2004). Subthreshold depression as a risk indicator for major depressive disorder: a systematic review of prospective studies. Acta Psychiatr Scand.

[B37] Haringsma R, Engels GI, Beekman AT, Spinhoven P (2004). The criterion validity of the Center for Epidemiological Studies Depression Scale (CES-D) in a sample of self-referred elders with depressive symptomatology. Int J Geriatr Psychiatry.

[B38] Bagby RM, Ryder AG, Schuller DR, Marshall MB (2004). The hamilton depression rating scale: has the gold standard become a lead weight?. Am J Psychiatry.

[B39] Nyklicek I, Scherders MJ, Pop VJ (2004). Multiple assessments of depressive symptoms as an index of depression in population-based samples. Psychiatry Res.

[B40] McAlpine DD, Wilson AR (2004). Screening for depression in primary care: what do we still need to know?. Depress Anxiety.

[B41] Lin CC, Bai Y, Chen JY (2003). Reliability of information provided by patients of a virtual psychiatric clinic. Psychiatr Serv.

[B42] Umefjord G, Petersson G, Hamberg K (2003). Reasons for consulting a doctor on the Internet: Web survey of users of an Ask the Doctor service. J Med Internet Res.

