# Supplementary material for: Web-based tools can be used reliably to detect patients with major depressive disorder and subsyndromal depressive symptoms
Source: BMC Psychiatry. 2007 Apr 10;7:12. doi: 10.1186/1471-244X-7-12 (PMC1855926; doi:10.1186/1471-244X-7-12)
Supplement: Additional file 1 — English translation of questions in the Internet-based Self-assessment Program for Depression (ISP-D). The English translation of ISP-D is preliminary and is intended for the ease of journal readers to understand the original Chinese version of ISP-D. [file 1471-244X-7-12-S1.doc]

English translation of ISP-D

The English translation of ISP-D is preliminary and is intended for the ease of journal readers to understand the original Chinese version of ISP-D. The translation is not validated. The wording of introduction has some differences from that used in the study period. The online complete description of the research project and informed consent form are not included.

**Index:**

| 這是集合多位國內外[資深精神科醫師](https://www.psychpark.org/guideline/list.asp)的專業所設計而成的網路自我評估系統，有鑑於[憂鬱症](http://www.psychpark.org/psy/special/depression.asp)將成為廿一世紀第二大疾病，如何更方便快速地早期診斷及治療，是憂鬱症防治上刻不容緩的事。目前民眾對於前往精神科就診仍有許多避諱，由於網際網路具有隱密方便的特性，我們希望能在網路上設計一個安全又符合國際專業標準的自我檢測系統，以方便民眾能早期偵測出憂鬱症。           憂鬱症自我檢測系統(ISPD)是國內第一個具人工智慧的憂鬱症系統，也是國際上少數以網路架構並進行臨床測試的系統之一，目前已初步完成臨床測試，並開放給有需要的網友使用。  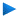[我要進行自我檢測](http://www.psychpark.org/guideline/depression/test/intro4.asp) |
| --- |
| The Internet-based self-assessment program was designed by the expertise of many senior certified psychiatrists. Depression will be the second burdensome illness in the 21the century. It is urgent to detect and treat depression as soon as possible. However, many people are still afraid of visiting a psychiatric clinic. Through the convenience of the Internet, we hope to design a private, internationally standardized self-assessment tool to help people find their depression in the early stage.  ISP-D is the first programmed depression system in Taiwan and one of the few Web-based depression systems ongoing clinical testing in the world. The preliminary clinical validation of the program is completed. It is open for whom need to test for depression.  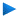[I want to perform the test](https://www.psychpark.org/guideline/researcher/ISPD/test/intro1.htm) |

Intro1

| 本系統已完成初步之研究測試，具有不錯的信度跟效度；在完成自我檢測後，若有需要請找精神科醫師評估，以確實了解自己得憂鬱症的可能性。        點【下一步】即開始檢測，之後會出現一問一答(選擇)的網頁，以下事項請注意:   - 請看清楚問題再回答，因為您選擇的答案是評估憂鬱症的重要參考資料，請務必提起精神，認真填寫哦!! - 若不懂問題的意思，可參考題中顏色稍淡的文字說明或舉例後再答。 - 若仍不懂題意，請在題目的最下方空格，填上您的疑問或建議，這樣我們可以在下一版時作修正，我們會感謝您提供意見。 - 若做到下一題時覺得上一題答案要修改，可點【上一步】重選。 - 如果開始進行檢測，表示您同意將資料儲存於心靈園地資料庫，且當您在心靈園地發問或求診時，允許回覆的專業人員查看您的檢測結果。   下一步 |
| --- |
| The system has completed the preliminary testing with fair reliability and validity. If you want to understand more about the possibility of getting depression, please find a psychiatrist after performing the ISP-D test.  Clicking “Next” button will begin the test. On most Web pages, you just need to click to choose the answer(s) for the corresponding question(s). Please pay attention to the following points:   - Please answer the questions only after reading them clearly. Your choice for each question is important for the evaluation of depressive symptoms. Please refresh your mind and answer the questions honestly so that you can achieve more accurate results. - If you don’t quite understand the meaning of a question, please read the interpretation or examples in different text color. - If you are still not clear about the meaning of a question, please fill your opinions or suggestions in the blank space underneath the answers. We thank for your precious opinions. - If you want to revise your answer of the last question, just click “previous” and re-choose your answer. - When you begin the test, it means that you agree to store the test results on the PsychPark database. If you ask a question in the virtual clinic of PsychPark in the future, the specialist responding to your question is allowed to see the test results.   Next |

First:

| 您覺得自己得到憂鬱症的可能性多高?【用您的直覺】  絕不可能 可能性不高，但我也不太確定 有可能，但我不太確定 我一定有憂鬱症 我不知道憂鬱症是什麼 |
| --- |
| How likely do you feel of getting depression?【By your intuition】  Never Not likely, but I am not sure. Possible, but I am not sure. I must get depression. I don’t know what depression is. |

S01:

| 您最近會不會時常覺得心情不好、或情緒低落？【譬如常覺得鬱卒、傷心、難過、沮喪、煩躁、害怕或容易發脾氣等】  會，一星期約有天會有這種感覺  不會 |
| --- |
| Do you often feel depressed or down recently? 【For example, being sad, unhappy, frustrated, fearful, or irritable frequently】  Yes, I have the feelings about days in one week.  No. |

S0101:

| 如果最近某一天出現這種心情不好或情緒低落的情形時，當天持續的時間會有多久？  幾乎整天  超過半天  小於半天  偶爾(一下子而已) |
| --- |
| How long did it last if the depressed or down feeling happened in a day?  almost all day  most of the day  less than half day  only for a while |

S0102:

| 整體而言，這樣心情不好或情緒低落的情形最近持續多久了？  小於二週  二週以上至半年  半年至二年  大於二年 |
| --- |
| Overall, how long has the depressed or down feeling persisted recently?  less than 2 weeks  2 weeks to half year  half year to 2 years  more than 2 years |

S0201:

| 您最近會不會幾乎整天對大部分的事物都感到失去興趣？   不會 會，小於二週 會，二週以上至半年 會，半年至二年 會，大於二年 |
| --- |
| Have you ever been much less interested in most things most of the time recently?   No. Yes, for less than 2 weeks. Yes, for 2 weeks to half year. Yes, for half year to 2 years. Yes, for more than 2 years. |

S0202:

| 您最近會不會幾乎整天對於以前覺得愉快的事物，都不再感到愉快了？   不會 會，小於二週 會，二週以上至半年 會，半年至二年 會，大於二年 |
| --- |
| Have you ever been much less able to enjoy the things you used to enjoy most of the time recently?   No. Yes, for less than 2 weeks. Yes, for 2 weeks to half year. Yes, for half year to 2 years. Yes, for more than 2 years. |

S03:

| 在沒有刻意增減體重的情形下，您的胃口或體重是否有明顯改變？【如體重在一個月內減少或增加5﹪】   否 是，小於二週 是，二週以上至半年 是，半年至二年 是，大於二年 |
| --- |
| Was your appetite or body weight decreased or increased without trying intentionally?【For example, by ±5% of body weight in a month.】   No. Yes, for less than 2 weeks. Yes, for 2 weeks to half year. Yes, for half year to 2 years. Yes, for more than 2 years. |

S04:

| 您是否幾乎每晚都睡不好？【可複選】  否 是，入睡困難 是，過早醒來，醒來後就睡不著 是，一個晚上睡睡醒醒好幾次 是，睡眠過多，但仍覺得睡不好 |
| --- |
| Did you have trouble sleeping nearly every night ?【You can choose several items】  No. Yes, difficulty falling asleep. Yes, early morning wakening.  Yes, waking up in the middle of the night. Yes, sleeping excessively. |

S0401:

| 這樣睡不好的情形出現的時間多久了？  小於二週  二週以上至半年  半年至二年  大於二年 |
| --- |
| How long have you trouble sleeping?  Less than 2 weeks. 2 weeks to half year. Half year to 2 years. More than 2 years. |

S05:

| 您是否自己覺得或曾有人告訴你有出現以下的情形? 【如：幾乎每天在說話、想事情、或行動時速度比平常緩慢，或者是比平常煩躁、無法平靜、或坐立不安？】   否 是，小於二週 是，二週以上至半年 是，半年至二年 是，大於二年 |
| --- |
| Did you feel or anyone tell you about the following condition?【Talk, think or move more slowly than normal or feel of fidgety, restless or having trouble sitting still almost every day?】   No. Yes, for less than 2 weeks. Yes, for 2 weeks to half year. Yes, for half year to 2 years. Yes, for more than 2 years. |

S06:

| 您是否幾乎每天都覺得疲倦、虛弱、或是沒有精神？   否  是，小於二週 是，二週以上至半年 是，半年至二年 是，大於二年 |
| --- |
| Did you feel tired, weak, or without energy almost every day?  No. Yes, for less than 2 weeks. Yes, for 2 weeks to half year. Yes, for half year to 2 years. Yes, for more than 2 years. |

S0701

| 您是否幾乎每天都覺得自己沒有信心或是沒有用？   否 是，小於二週 是，二週以上至半年 是，半年至二年 是，大於二年 |
| --- |
| Did you feel worthless or lack of confidence almost every day?  No. Yes, for less than 2 weeks. Yes, for 2 weeks to half year. Yes, for half year to 2 years. Yes, for more than 2 years. |

S0702:

| 您是否幾乎每天批評自己，覺得自己做錯事，或對不起別人？  否 是，小於二週 是，二週以上至半年 是，半年至二年 是，大於二年 |
| --- |
| Did you criticize yourself, feel guilty or doing something wrong almost every day? No. Yes, for less than 2 weeks. Yes, for 2 weeks to half year. Yes, for half year to 2 years. Yes, for more than 2 years. |

S08:

| 您是否幾乎每天都覺得記憶力不好、不專心、或是猶豫不決？  否 是，小於二週 是，二週以上至半年 是，半年至二年 是，大於二年 |
| --- |
| Did you have poor memory, difficulty concentrating or making decisions almost every day? No. Yes, for less than 2 weeks. Yes, for 2 weeks to half year. Yes, for half year to 2 years. Yes, for more than 2 years. |

S09:

| 您是否幾乎每天都會覺得想不開、活著不值得、或甚至想死？  否 是，小於二週 是，二週以上至半年 是，半年至二年 是，大於二年 |
| --- |
| Did you think of death, feel life is not worth living, or wish that you were dead almost every day?  No. Yes, for less than 2 weeks. Yes, for 2 weeks to half year. Yes, for half year to 2 years. Yes, for more than 2 years. |

Fun01:

| 過去兩週上述的症狀對您與朋友、同事或是家人的關係造成的影響有多嚴重？  完全沒有 稍微 (或偶爾會影響) 明顯 (或常常會影響) 很嚴重 (或一直會影響) |
| --- |
| How did the above symptoms impair your relationship with your friends, colleagues, or family members?  Not at all. Mildly (or occasionally) Moderately (or often) Severely (or always) |

Fun02:

| 過去兩週上述的症狀對您的學業或是工作造成的影響有多嚴重？  完全沒有 稍微 (或偶爾會影響) 明顯 (或常常會影響) 很嚴重 (或一直會影響) |
| --- |
| How did the above symptoms impair your ability to function at work or academic performance?  Not at all. Mildly (or occasionally) Moderately (or often) Severely (or always) |

Fun03:

| 過去兩週上述的症狀對您處理日常生活事務﹝如：作家事、購物等﹞造成的影響有多嚴重？  完全沒有 稍微 (或偶爾會影響) 明顯 (或常常會影響) 很嚴重 (或一直會影響) |
| --- |
| How did the above symptoms impair your ability to perform activities of daily living?  Not at all. Mildly (or occasionally) Moderately (or often) Severely (or always) |

Med:

| 您目前是否有重大身體疾病或慢性病﹝如：糖尿病、高血壓、腦中風等疾病﹞？   有〈請繼續作答下列問題〉     沒有〈請直接點下一步〉   |  | | --- | | 如果有重大身體疾病或慢性病，您的疾病診斷是什麼？ | | 目前是否因此疾病有在服用藥物？   是                   否 | |  | |
| --- | --- | --- | --- | --- |
| Do you have any major physical illness or chronic illness now (For example, diabetes mellitus, hypertension, or stroke et al.)?  Yes (please continue to answer the next question).  No (please click the “next” button) |
| What’s the diagnosis of your physical illness? |
| Do you take medicines for the disease?   Yes                  No |

Abuse:

| 您最近是否有喝酒或使用可能影響精神的藥物【如：安非他命、搖頭丸、FM2、大麻等】？   是  否 |
| --- |
| Did you drink wine or use any substance that might affect your mental status? (eg. Amphetamine, MNDA, FM2, marijuana et al)    Yes  No |

Bere:

| 您最近是否有家人或是親近的朋友過世？   是  否 |
| --- |
| Did any of your family members or close friends pass away recently?  Yes  No |

Psych01:

| 您最近是否常會聽到一些說話的聲音，但是旁人都聽不到？【譬如：身旁沒有人，但卻聽到有人在跟自己說話】【耳鳴等嗡嗡聲不算，內心或腦子在想的話不算】   是  否 |
| --- |
| Did you often hear some voices, which others can not hear of? (For example, hearing of voices spoken to you but nobody around you.) (The buzz of tinnitus and the inner thoughts are not included.)   Yes  No |

Psych02:

| 您最近是否常會覺得有人要對你不利【如：跟蹤你、迫害你或是想要控制你】？   是  否 |
| --- |
| Did you often feel that somebody would do something harmful to you? (For example, follow you, persecute you, or want to control you.)    Yes  No |

Mania:

| 您是否曾經有躁症的病史【如：不尋常的情緒高昂、多話、精力旺盛或是活動力增加，持續一星期以上】？   是  否 |
| --- |
| Did you have history of manic symptoms? (For example, unusually elated mood, hypertalkative, energetic, or excessive activities lasting for more than one week?)  Yes  No |
